# Process and Product in Cross-Cultural Treatment Research: Development of a Culturally Sensitive Women-Centered Substance Use Intervention in Georgia

**DOI:** 10.1155/2014/163603

**Published:** 2014-09-22

**Authors:** Hendrée E. Jones, Irma Kirtadze, David Otiashvili, Kevin E. O'Grady, Keryn Murphy, William Zule, Evgeny Krupitsky, Wendee M. Wechsberg

**Affiliations:** ^1^UNC Horizons and Department of Obstetrics and Gynecology, School of Medicine, University of North Carolina at Chapel Hill, 127 Kingston Drive, Chapel Hill, NC 27514, USA; ^2^Departments of Psychiatry and Behavioral Sciences and Obstetrics and Gynecology, School of Medicine, Johns Hopkins University, Baltimore, MD 21224, USA; ^3^Addiction Research Center, Alternative Georgia, 0177 Tbilisi, Georgia; ^4^Ilia State University, School of Arts and Science, 0162 Tbilisi, Georgia; ^5^Department of Psychology, University of Maryland, College Park, College Park, MD 20742, USA; ^6^Substance Abuse Treatment Evaluations and Interventions Research Program, RTI International, Research Triangle Park, NC 27709, USA; ^7^Department of Addictions, Bekhterev Research Psychoneurological Institute, 192019 Saint Petersburg, Russia

## Abstract

Women who inject drugs (WID) are highly marginalized and stigmatized and experience ongoing discrimination in Georgia. Few opportunities exist for WID to receive publicly funded treatment for substance use disorders. The IMEDI (Investigating Methods for Enhancing Development in Individuals) project was developed in response to the need for women-specific and women-centered treatment services. This paper described our approach to understanding the Georgian culture—and WID within that culture—so that we could integrate two interventions for substance use found effective in other Western and non-Western cultures and to outline how we refined and adapted our integrated intervention to yield a comprehensive women-centered intervention for substance use. Reinforcement Based Treatment (RBT) and the Women's CoOp (WC) were adapted and refined based on in-depth interviews with WID (*N* = 55) and providers of health services (*N* = 34) to such women and focus groups [2 with WID (*N* = 15) and 2 with health service providers (*N* = 12)]. The resulting comprehensive women-centered intervention, RBT+WC, was then pretested and further refined in a sample of 20 WID. Results indicated positive pre-post changes in urine screening results and perceived needs for both RBT+WC and a case management control condition. The approach to treatment adaptation and the revised elements of RBT+WC are presented and discussed.

## 1. Background and Aims

Injection-drug-using individuals are highly marginalized, highly stigmatized, and are at increased risk for STIs, HCV, and HIV worldwide [1]. Women who inject drugs (WID) experience this same marginalization, stigmatization, and increased risk for disease—without any recognition for the need for research and service delivery focused on their unique needs [1]. WID in Georgia are likewise highly marginalized and stigmatized and experience ongoing discrimination in Georgia. Although they represent up to 10% of the adult substance-using population [2, 3], only 1–5% of drug-related service beneficiaries are women [4, 5]. The World Health Organization [1] has recently called for worldwide efforts to provide treatment services for women to meet their unique needs, including physical abuse and violence.

Women in Georgia who use illicit substances commonly experience emotional abuse, physical aggression, and sexual violence [6]. Such violations are rooted in social norms and traditions and a cultural environment that supports asymmetry in gender roles and places restrictions on women's freedom and independence [7]. Recent economic problems in Georgia have facilitated women's rise in societal importance through increased employment opportunities; however, men often respond to their perceived loss of power by reaffirming their positions through drinking and physical force [8].

WID are at high risk for contracting and spreading HIV [9]. Injection drug use impairs judgment and thereby increases risky sex behaviors, including unprotected sex and having multiple sex partners. Sharing contaminated injection equipment and sexual contact are also major causes of HIV infection in WID in countries such as Georgia whose injection-drug-using populations are at high risk for HIV and hepatitis C virus (HCV) infections [10]. Moreover, the power inequality experienced by WID in heterosexual relationships raises the HIV infection risk, because the loss of power lessens their ability to negotiate safer sex practices out of fear of abuse by their sex partner or the need to meet basic survival needs such as food and shelter [11]. However, empirical research regarding the reasons for HIV transmission and the nature and extent of physical abuse and sexual violence in WID in Georgia is lacking.

There are few opportunities for women to receive publicly funded substance use disorder treatment in Georgia. Given that drug treatment has been designed to serve male beneficiaries—when treatment is available—it lacks sensitivity to the unique needs and challenges that WID face in their daily lives [12, 13].

The purpose of the present paper is to present the methods and findings of a study whose purpose was the development and pilot testing of a culturally sensitive, women-centered intervention for WID in Georgia. This section introduces the overall project and describes our first two studies, which represent our approach to understanding the Georgian culture—and WID within that culture—in order that we could integrate two interventions for substance use found effective in other Western and non-Western cultures. The Materials and Methods section then outlines the general methodological approach of the project and how we refined and adapted our integrated intervention to yield a comprehensive women-centered intervention for substance use. Results present a summary of qualitative and quantitative findings from the pilot projects, while Discussion reviews the “lessons learned” from the pilot study.

### 1.1. The IMEDI Project

The IMEDI (Investigating Methods for Enhancing Development in Individuals) project was developed in response to the need for women-specific and women-centered treatment services. IMEDI is the Georgian word for “hope”, the feeling we aimed to give women participating in the project. The IMEDI project has two goals: first, to gain the necessary knowledge from WID and treatment providers about drug use, HIV risk behaviors, and the current drug treatment in Georgia, second, use this information to adapt, integrate, and implement a comprehensive treatment program to slow HIV and HCV transmission in Georgia.

#### 1.1.1. Project Design

**Figure 1:**
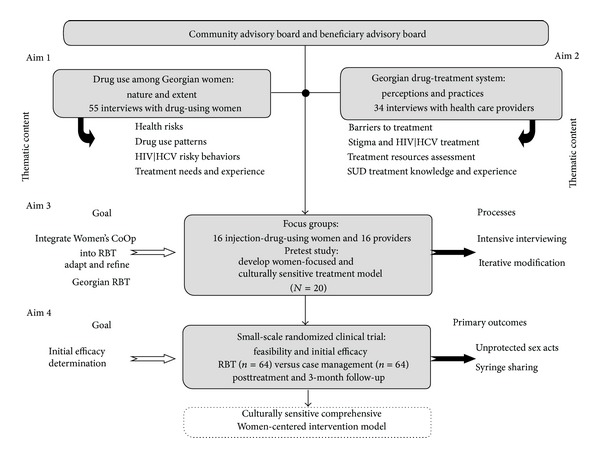
Overall design of the IMEDI project.

The overall IMEDI Project design is depicted in Figure 1. This figure reflects the final study design rather than the initial study design. We note below how the study design changed as the project progressed and how those changes are reflected in the figure.

Figure 1 also reflects on both the process and product of the project. Reading from the bottom up, the ultimate outcome of the project was a culturally sensitive comprehensive women-centered intervention for Georgian WID. Reading from the top down, the goals of the project can be seen to have 4 aims corresponding to 4 different studies, hereinafter referred to as Aim *n* study, with *n* corresponding to the particular aim. Aims 1 and 2 involved the conduct of two separate qualitative interview studies of WID and the health care providers. The results of these analyses were reviewed by our Advisory Boards, which then fed into the initial development of our culturally sensitive, comprehensive, women-centered intervention for substance use. The Aim 3 study involved the cyclical refinement and adaption of this intervention, and findings from this study are the primary foci of the present paper.

#### 1.1.2. Aim 1 Study: Findings

In-depth semistructured interviews 60–120 minutes in length were conducted with 55 WID [6]. Several recurring themes appeared across the interviews. First, women in Georgia experience high levels of guilt and shame as a result of their substance use. Second, both unsafe injection practices and unsafe sexual contacts result in frequent exposure to blood-borne and sexually transmitted infections by substance-using women. Third, the use of condoms is often seen as a violation of social norms; moreover, they are viewed as a method of birth control not as a means for HIV/STI prevention. Fourth, emotional abuse is more common than physical violence against WID. Their sexual partners frequently perceive emotional abuse as more effective than physical violence. The women strongly desired a trusted person from whom they could receive counselling and the desire for skills to improve their health and well-being and in order to overcome their isolation, boredom, and fear. Fifth, although no woman interviewed indicated they had done so, participants indicated that exchanging sex for drugs and commercial sex work were frequent practices engaged in by WID in Georgia, who were said to travel to Turkey to engage in such work. Finally, women infrequently received treatment for their substance use disorder due to fear of repercussions from social, legal, employment, and medical people in their life and the lack of availability of either women-sensitive or women-focused treatment services. Seeking help for a substance use disorder was seen as possibly undermining their success as women, which is defined culturally only in terms of their roles as daughter, wife, and mother. However, the need for women-focused substance use treatment services in Georgia was stressed by almost all participants.

#### 1.1.3. Aim 2 Study: Findings

In-depth semistructured interviews 60–120 minutes in length were completed with 34 health service providers who had previously provided services to a WID one or more times in the two months prior to the interview [14]. Results indicated that medication-assisted withdrawal (i.e., detoxification) was the predominant treatment for substance dependence in Georgia and providers believed that opioid agonist medication yielded superior treatment outcomes in comparison to medication-assisted withdrawal. Providers had less tolerance towards WID than men who inject drugs, saw WID as having more severe problems than their male counterparts, and predominantly believed that substance-using women were failures as mothers, wives, and/or daughters. Most providers were unaware of the availability of specific types of drug-treatment services in their city and did not seek connections with other service providers, indicating a lack of linkages between drug-related services and other services. Two important points emerged from these interviews. First, a comprehensive network of service linkages for all patients in substance use treatment would likely markedly improve such treatment in Georgia. Second, there is a critical need in Georgia for women-specific substance-abuse services.

## 2. Materials and Methods

### 2.1. Overview

Because of their importance, both to the overall project and to our Aim 3 study, this section describes our Community Advisory Board and our Beneficiary Advisory Board, prior to a discussion of the Aim 3 study methods. Moreover, general issues germane to Aim 1 and/or Aim 2 studies and to our Aim 3 study are described in this section.

All three studies were approved by the Office of Research Protection Institutional Review Board (IRB) at RTI International, USA, and the IRB at the Maternal and Child Care Union, Georgia.

### 2.2. Advisory Boards

#### 2.2.1. Community Advisory Board (CAB)

Our CAB, chaired by our Georgian principal investigator (IK), met 2 times per year during the intervention development phases. The CAB assisted the investigative team in several ways, most notably in understanding stigma and barriers to treatment and then developing an infrastructure of organizations that would serve as a lasting and sustainable contribution to improving the network of services provided to WID in Georgia. CAB members include local experts in women's health and services that are provided to WID, drug treatment providers, professionals working in prisons and law enforcement, women's violence intervention, providers of family planning and other aspects of women's reproductive health, counselors that provide HIV and STI counseling, HIV prevention services, women in the Global Fund's Country Coordinating Mechanism who oversee HIV and tuberculosis prevention and treatment programs in Georgia, and a methadone maintenance treatment facility director (*N* = 11).

During the opening stages of the project, the CAB aided the investigative team in identifying and establishing an outreach presence in several cities in Georgia. Moreover, the CAB provided input and feedback on the development of the interview guides for Studies 1 and 2. Finally, it provided input, guidance, and feedback on the interpretation of the interviews. Its role in our Aim 3 study is outlined below.

#### 2.2.2. Beneficiary Advisory Board (BAB)

The BAB was created to allow input from drug-using women into all project aspects. The BAB included 4 WID in Georgia who provided their knowledge and experience to the project. At the time of development, there were 200 patients currently in opioid agonist treatment in a partner clinic, 2 of which were women. One opioid agonist-treated woman described being harassed by the police and the absence of women-centered treatment for women in Georgia. She liked our project and agreed to help us recruit other WID for a woman-centered intervention. The BAB provided their commentary and feedback regarding the study recruitment and intervention materials and interpretation of findings.

### 2.3. Recruitment

WID in Aim 1 and Aim 3 studies were recruited via low threshold programs using referrals from our CAB/BAB and snowball sampling. Health service providers in our Aim 2 study were recruited from a list of health care providers provided to the project staff from the CAB who thought the individuals in question might meet study criteria.

Potential participants met with project staff who outlined the particular study and collected initial eligibility information. Potential participants who expressed an interest in the study made an appointment with project staff to meet at a mutually convenient time and at a private location, at which time consent for participation in the interview or focus group was obtained; or, in the case of the Aim 3 study, participation in the small-scale pilot study was secured.

All participants provided written informed consent prior to participation.

No participant entered more than a single project study; that is, no Aim 1 study participant entered either Aim 3 or Aim 4 study, and no participant in Aim 3 study entered Aim 4 study.

### 2.4. Interviews and Focus Groups

All focus groups (and the individual interviews in our Aim 1 and Aim 2 studies) were facilitated by a senior project researcher (IK), who had considerable prior experience in conducting focus groups for research purposes and was extremely knowledgeable regarding qualitative research methods. All interviews were audio-recorded, with the prior written consent of participants.

### 2.5. Qualitative Analysis

Qualitative analyses for Aims 1, 2, and 3 studies followed the same general procedure. Audio recordings were transcribed directly into Georgian in Unicode text format. Transcripts of these text files were exported into PDF files, which were then imported into nVivo 9 (http://www.qsrinternational.com/products_nvivo.aspx) qualitative analysis software. Content and thematic analyses were then conducted under the direction of IK. Two coders independently coded relevant textual material from the recordings. nVivo was used to search the text for themes that were then coded, examined, and collated to form subcategories and categories. The general focus of the analysis in each study was on the opinions, attitudes, beliefs, and/or behavior of the participants, which varied by study.

### 2.6. Study 3: Small-Scale Pilot Study

#### 2.6.1. Participants

WID (*N* = 20) who met eligibility criteria (conversant in Georgian, able to provide informed consent, minimum 18 years of age, injection of illicit drugs in the past 30 days as verified by venipuncture stigmata, and sexually active at least once in the past 30 days) and who were seeking treatment were recruited to participate in drug treatment research, with block randomization of each successive pair of participants to either one or the other treatment condition, such that *n* = 10 in each condition.

#### 2.6.2. Recruitment and Field Screening

Standardized street outreach techniques were used to recruit WID from outreach sites identified by our CAB and BAB in Tbilisi and Gori. Outreach workers used venue-based sampling methods that were adapted to fit the conditions in each city and each site. Outreach workers were trained in the recruitment procedures and protocols and the recruitment procedure was scripted in a manual. Essentially, outreach workers recruited WID by talking to them in the specified venue locations and by distributing brochures describing the study. A field screening instrument was used to make the initial determination of eligibility and refer potential participants to the study field office for the final determination. No personal identifiers were recorded at this time; however, WID who met initial eligibility criteria were given referral ID numbers that would become their study identification number if they are enrolled in the study.

#### 2.6.3. Study Site

A site with sufficient office and research spaced was rented for the Aim 3 study in the Saburtalo district of Tbilisi. This space had no previous affiliation with substance use or HIV prevention services, thus reducing to the minimum possible barriers for women to visit the site.

#### 2.6.4. Development of the Interventions

*Overview.* Findings from the Aims 1 and 2 studies were instrumental in adapting and revising the comprehensive women-centered intervention, as well as refining the case management condition adaptation. Efforts proceeded in six stages: (1) adapting the US based case management manual and materials; (2) integrating the modules from the original Women's CoOp and other editions such as the Women's Health CoOp South Africa and the Russian Women's CoOp [15, 16] into Reinforcement Based Treatment (RBT) [17] to create a comprehensive women-centered intervention, RBT+WC; (3) reviewing and critiquing of the RBT+WC and case management manuals and materials by our CAB and BAB, followed by revisions of such materials based on CAB and BAB feedback; (4) focus groups comprised of either active WID or providers who likewise reviewed and critiqued the RBT+WC and case management manuals and materials; (5) reviewing outcome measures and possible instrumentation issues; and (6) recruiting active WID interested in treatment who were randomized to either the RBT+WC or case management condition. Because we were actively adapting and refining both interventions during this stage of the project, pilot-testing was undertaken in blocks of participants who were interviewed either during treatment or at treatment completion, in order to make modifications after each block of participants completed treatment.

Based on findings from the Aims 1 and 2 studies [6, 14, 18], we anticipated that our participants would have multiple medical, legal, psychiatric, financial, behavioral, and social service problems including HIV risks. Accordingly, we developed our intervention and case management conditions to address these potential issues.

*Case Management Condition.* Development of a case management condition was necessary because there were* no* available active control conditions in Georgia for substance-using women against which RBT+WC could be compared.

The general focus of the case managers in the case management condition was to first identify the needs and severity and acuity of the needs women have using a standardized needs assessment instrument. Next, the case manager worked with the participant using a comprehensive resource guide developed with the input of our CAB and BAB that identified services and programs where the participant might be eligible to receive services. The case manager then provided instructions about how to contact those resources. The case manager role-played with the participant about how to contact the service and what to say and what not to say. The case manager also worked with the participant on determining the barriers the participant might face in accessing that service and how to minimize or overcome such barriers. At each meeting, the case manager and the participant reviewed the progress that had been made in accessing the service and the need for reprioritizing the plan for service access (e.g., housing may have been found but then the participant lost her housing, making housing an acute need again). A plan was then made in terms of the order in which the services would be contacted and sought. A phone was provided and minimal funds for transportation were given on specified days to support the participant in accessing services.

*Development of RBT+WC.* In reviewing the available behavioral interventions that have been tested with women, Reinforcement Based Treatment (RBT) appeared to be a promising intervention to adapt for women living in Georgia. RBT reduces drug use and related injection-drug-use risk behaviors in both men and women [17, 19]. RBT is a social-learning-theory-driven, evidence-based drug treatment intervention that employs life skills training, recreational therapy, and employment as components of a comprehensive treatment model [20]. Other strengths of RBT also make it a promising, desirable, and culturally compatible treatment approach for female Georgians. For example, Georgians are extremely family-oriented, and RBT makes efficient use of the family and social support structure of the participant to reinforce drug abstinence. Georgians are typically nonconfrontational in their communication and interaction styles; thus, the reliance on a motivational interviewing style to guide all participant-counselor interactions will work extremely well in fostering a positive therapeutic relationship. Typical Georgians are well educated, highly literate, and industrious, which fits well with the RBT employment goal. Finally, RBT takes a proactive and concurrent approach to addressing the multiple needs of participants, a required treatment component needed for addressing the complex life issues of WID in Georgia.

Like many substance abuse treatments, RBT effectively reduces injection drug use and related risk behaviors but not sexual risk behaviors. This lack of effect may be in part because it does not include specific skills to promote condom competence or communication skills to negotiate condom protection. Thus, incorporated into RBT was a component to address women-centered sexual risk behaviors based upon selected treatment modules from the Russian Women's CoOp intervention (WC) [21], and adapted to the RBT+WC for the cultural context of Georgian WID.

WC is considered a best-evidence intervention and has been adapted in other countries and found efficaious [16]. Based in feminist theory and empowerment theory [22] and principles of social cognitive theory [23], the goal of Women's CoOp is about educating regarding substance use and abuse, sexual risk, and gender-based violence and reducing risk behaviors by helping to develop assertive skills and a personal concrete harm reduction plan within a socially supportive environment [24].

**Figure 2:**
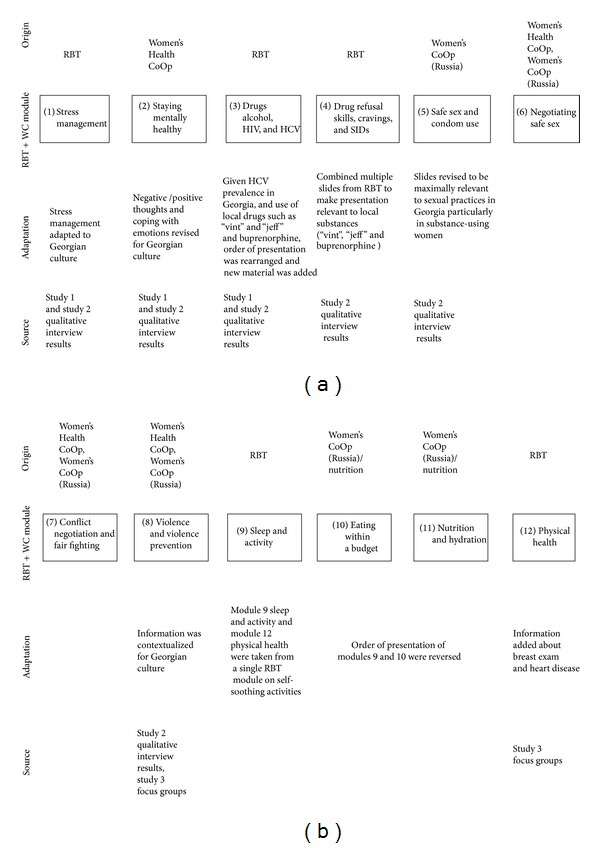
RBT+WC intervention modules: source and adaptation.

RBT and WC have complementary strengths that had the potential to yield a comprehensive, women-centered intervention. However, it was necessary to revise both RBT and WC to produce an intervention that would be sensitive to the values and context found in the Georgian culture.  Figure 2 illustrates the process of change that was undertaken to refine and adapt the RBT and WC treatment modules to produce RBT+WC. In developing RBT+WC, usual formative steps were used to help inform the contextual and cultural nuances necessary to address their risk and challenges in addition to the integration of the selected evidence-based interventions [25, 26], with particular attention to the 5 steps outlined by McKleroy et al. [25].

### 2.7. Feedback from the CAB and BAB

Both the BAB and CAB reviewed the treatment manuals. Important feedback common to both groups included a greater focus on communication skills-building, the need to build a trusting relationship with the women, and a greater focus on the employment and financial needs and empowerment of the women.

### 2.8. Focus Groups

Prior to conducting our pilot feasibility study, we conducted four focus groups, two with active WID (*N* = 15 total) and two with health care providers (*N* = 12 total) participants, respectively. In each focus group, the materials related to the RBT+WC and case management condition were reviewed for accuracy and appeal of content and presentation and lack of stigmatizing language. Participants reacted to and commented upon both strengths and weaknesses of the materials. For the providers, we sought to examine to what extent these materials would be practical and useful for them to use in the future and what changes would need to be made to achieve that goal. The order of discussion of the two models was counterbalanced across the respective types of groups. The goal of these focus groups was to inform further refinement and adaptation of the two interventions to better address the needs of Georgian WID.

Tailoring RBT+WC to yield a culturally sensitive women-center intervention for WID in Georgia, the qualitative findings from the Aims 1 and 2 studies were discussed by the US-Georgian research team and Russian collaborator Dr. Evgeny Krupitsky. For this purpose IK prepared summary of the findings from the interviews as illustrated by appropriate quotes, in most of the cases in the form of tables, and additionally supported by a measure of degree of saturation. Visual displays such as word frequency query and connection mapping of thematic results were used to identify major issues and needs of women. This process meaningfully informed the identification and prioritization of topics for intervention modules with the following ones selected as foci for the intervention: stress; mental health; physical health; drug and alcohol use; craving; STIs, HCV, and HIV; safe sex and condom use; negotiating safe sex; conflict negotiation; and violence prevention.

Our goal in tailoring RBT and WC to create RBT+WC was to produce an intervention that was optimal for this population. Our belief is that many if not all populations of WID face similar problems, such as stigma and abuse. Thus, the goal in adapting RBT+WC to Georgian culture was to revise the RBT+WC intervention so that it used the language of the culture, so that its content and process were understandable to the treatment population, and its goals were consistent with the goals of women entering the treatment. Thus, revision of RBT+WC was guided by a desire to maximize its treatment relevance and credibility, and in doing so, maximizing its efficacy. This focus on the cultural sensitivity and ecological validity of RBT+WC is consistent with recommendations on cultural adaptations of interventions [27, 28].

### 2.9. Outcome Measures

A revised version of the Risk Behavior Assessment (RRBA) developed for research in Russia served as the primary outcome measure [21]. The RRBA combines measures used in earlier studies and has 10 sections that contain questions about demographics and social characteristics, health knowledge, alcohol use, drug use, drug injecting, sexual practices, power and empowerment, conflict and victimization, physical and mental health, and HIV status. The principal questions of interest include those questions regarding drug injection and sexual practices. In addition, urine specimens were collected during the entire 6-week treatment under direct observation of trained staff, twice weekly with one group of participants and thrice weekly for the remaining groups of participants, to determine the most acceptable schedule for urine drug monitoring. We used tests manufactured by ACON Laboratories, Inc. The test-strip device simultaneously detected the presence of opiates (100 ng/mL cutoff level), buprenorphine (10 ng/mL cutoff level), methadone (200 ng/mL cutoff level), THC (25200 ng/mL cutoff level), amphetamine and methamphetamine (300 ng/mL cutoff level), and benzodiazepines (100 ng/mL cutoff level). Each time a urine sample was collected, a breathalyzer reading was also obtained.

Based on findings from our Aim 1 and Aim 3 studies, the RRBA was supplemented with a time-line follow back (TLFB) procedure to assess both substance use and sex-risk behaviors. The TLFB procedure was intended to provide information of drug use and sexual activity weekly throughout the participant's enrollment in the study, rather than simply at the beginning and end of treatment. We modified the TLFB [29, 30] for use in our population.

### 2.10. Instrumentation and Measurement Issues

The RRBA has been used in international WC outcome research for more than 10 years and has been translated from English into several other languages, including Russian. Moreover, three members of the investigative team were responsible for the earlier pilot research in Georgia and were familiar with issues regarding cultural sensitivity of the measures and their translation and back-translation. Two members of the investigative team were from Georgia and were fluent in reading and writing both Georgian and English, as were the project manager and research staff. Thus, the RRBA was reviewed in English and revised in English in a series of meetings with project staff in English so the English phrasing of the measure could be accurately translated into Georgian so that it would accurately assess substance use and risky behavior in our population. The RRBA was then translated into Georgian, back-translated, and then reviewed by Georgian staff for accuracy and completeness in translation.

### 2.11. Cyclical Process for the Adaptation and Refinement of RBT and Case Management

Both baseline and post-treatment-completion data were collected. Each participant was intensively interviewed according to an adaptation and refinement process that proceeded in three cycles. After each cycle, the RBT and case management interventions were refined as necessary, and the respective interventions provided to the next cycle (the exception being the last cycle, after which the two interventions were adapted and revised a final time in preparation for the small-scale randomized clinical trial as part of the Aim 4 study). The first two cycles involved 3 participants from each of the two treatments, while the last cycle involved 4 participants from each of the two treatments. In each cycle, one participant was interviewed after each week of treatment, one was interviewed after each 4 weeks of treatment, and one was interviewed only at the conclusion of treatment; in the final cycle, there was an additional participant in each treatment who was interviewed only at the conclusion of treatment. The goal of this interview schedule was to obtain a “snapshot” of participants' views of treatment and the treatment process at different points in treatment. Although it might be argued that all participants should be interviewed weekly, such frequent interviews “sensitize” participants to treatment and the treatment components. Thus feedback from such participants would be unrepresentative of how the treatment would be viewed by someone who was provided the treatment without such interview schedule. Therefore, we designed our interview schedule to minimize the possibility by interviewing participants at various points in treatment.

## 3. Results

### 3.1. Focus Groups

Quantitative data were collected as part of the Aim 3 study to determine possible issues with measures and to assess the need to make changes in the treatment process. As with Aims 1 and 2 studies, findings from the focus groups were reviewed by our CAB and BAB. The two groups met separately and first reviewed a summary of qualitative findings from Aims 1 and 2 studies. This was followed by discussion of recreational activities to be offered to study participants. CAB and BAB members proposed a wide range of both recreational and skills-building activities, such as swimming, manicure/pedicure, hair cutting/dyeing and skin care courses, automobile driving schools (few women in Georgia have driver's licenses), and pastry cooking courses. Based on these recommendations and discussion within the project team, two activities—beads working and felt making—were selected for the project. Moreover, both the CAB and BAB provided important feedback regarding the order of topics for intervention modules to be delivered to participants (see Figure 2).

### 3.2. Participants

Participants in the Aim 3 study were all Georgian citizens, one native Russian and another native Chechen, the remaining were native Georgian.  Table 1 summarizes the demographic and background characteristics of the sample. Mean age was 34 years (SD = 10); 12 (60%) had obtained a vocational education (2 years in vocational or community college after high school) or a university degree; 13 were unemployed; and 12 (60%) lived with their main sex partner. The majority of women had experienced psychological problems at some point in their lives; many had been subject to physical and sexual violence. They were generally sexually active (all women had engaged in sexual intercourse at least once in the past 30 days), although the use of condoms was relatively infrequent. Main drugs injected at intake were opioids and homemade amphetamines/methamphetamines.

### 3.3. Outcomes

Two variables that we thought would be informative regarding the efficacy of the intervention were chosen as primary outcomes. The first is the results of the weekly urine screening test illicit drugs. Given the small sample size and the sparseness of the data on weekly evaluation of each drug under assay, we chose to examine this outcome at baseline (prior to the beginning of the intervention) and at the end of the intervention, as a dichotomous variable, with yes for a positive result for any illicit drug and no otherwise. The second outcome was the results of a needs assessment that is part of the RRBA, and so needs assessment data were collected at baseline and at 12 weeks at the end of the intervention, thus matching with the assessment time points for the first outcome. The needs assessment asks the participant to indicate whether she felt she needed help with any of the 20 issues (e.g., “help with getting out of debt or managing money,” “help with psychological services,” and “help with getting medical/healthcare services). Again, due to the sparseness of responding to each question, a single needs assessment score was determined by summing the yes responses to the 20 questions.

Both outcomes were examined with generalized estimating equations (GEE) models, with the between-subjects effect of intervention condition, the within-subjects effect of assessment time point, and their interaction, with biological assay for illicit drugs assumed to be a binary variable following a binomial distribution, and the needs assessment assumed to be a count variable following a Poisson distribution.

Table 2 contains the means and standard errors for these two outcomes for the two main effects and the interaction. For both variables, there was significant constructive change from baseline to end of treatment (*P* < 0.04 and *P* < 0.001, resp.), with positive urine screening test results declining by 50% and the number of perceived needs on the part of the participants declining more than 60%. Neither the main effect for intervention condition nor the interaction effect was significant for either outcome (all *P*s > 0.1).

## 4. Discussion

We were gratified to find that our participants reported constructive change in their lives as a function of treatment, at least in terms of the two outcomes we examined. Although we might have hoped to find a significant interaction that favored RBT, we doubted that we had any reasonable power to detect an interaction effect in a pilot study whose primary goals were to establish feasibility and acceptance of treatment on the part of the participant population. In that regard, we did conclude that our pilot study was a success, with only one participant dropping out of treatment prior to session 12, from the RBT condition.

Moreover, we did reach a number of conclusions from this study that we will apply to the Aim 4 study. First, in terms of measures, the TLFB did not seem to be informative above and beyond the information collected by the RRBA, so we have decided to omit it from use in study 4. Second, many items on the RRBA had a low frequency of responding (e.g., physical violence questions), so the RRBA was streamlined in order to assess outcomes directly relevant to the intervention conditions. Third, in order to maximize continuing contact with participants, rather than offering the intervention once per week over a 12-week period, it was decided to offer it twice per week for 6 weeks in our ongoing Aim 4 study.

Finally, the incentive structure for participation was substantially revised. The amount and form of incentives were discussed with the CAB and BAB and with focus groups during interviews and at the initial stage of the research. Based on these discussions participants were offered the cash-equivalent of 15 USD in local currency (Georgian GEL) for every visit and the equivalent of 10 USD for every breath and urine sample provided. In addition, in the case of RBT condition, in order to encourage abstinence, these samples had to be negative for monitored substances. However, feedback from study participants in this regard was critical, based on the perceived inequality between the two conditions. Thus, provision of monetary incentives for participation in the study was revised such that participants received the equivalent of 20 USD for completing the baseline and end-of-treatment interviews and 10 USD for each completed interview following sessions 1–12, with an additional 5 USD for providing breath and urine samples at each of these visits. Therefore, participants were able to receive a maximum of 220 USD (362 GEL) in incentives for participation, and payment in the RBT+WC was not contingent on provision of a negative urine sample. We stress that payment to participants were for their participation in the study and not as motivation for behavior change. It may be of considerable interest to pair RBT+WC with contingency management in future research.

*Limitations.* As with any small-scale pilot study, the present study has several notable limitations. The first limitation is that the study was not powered to detect differences between the treatment conditions, and so any conclusions reached in regard to the relative efficacy of either treatment cannot yet be drawn. Second, the extent to which the sample reflects the larger population of WID in Georgia is unknown. Clearly, the sample is reasonably well educated, and most appeared to be in stable relationships. However, the extent to which such circumstances are unrepresentative of WID in Georgia needs further research. Third, although participants completed the RRBA at baseline and after treatment, they were interviewed at various points during treatment, as noted above. They were specifically asked for their comments on the RRBA after the baseline assessment. Therefore, we believe that the responses to the follow-up measures have been contaminated to some degree by the interview process, which may have attuned participants to attend to the assessment measures and process. Fourth, our choice to discontinue use of the TLFB may have been premature and may reflect more on the specifics of the current sample and the participants' limited range of sexual activity. It may be that the TLFB would be quite informative, particularly if we would have put greater emphasis on obtaining information regarding unprotected sex. Finally, although Aim 3 study focused on the development of a comprehensive, culturally sensitive, and women-centered intervention, we understand that such an intervention may be necessary but not sufficient to effect behavior change in WID in Georgia. There are likewise changes that need to be effected in the larger treatment system, including policies of the Ministry of Health. Otiashvili et al. view the treatment system in Georgia from a policy perspective and detail the changes that need to be undertaken by the larger treatment system for any treatment of WID in Georgia to optimally effect change [31].

## 5. Conclusions

This series of studies is the first of its kind for reaching substance-using women in Georgia. It was an iterative process with many partners who all offered important additions that helped create a more comprehensive and culturally congruent intervention. One vitally important lesson that we had learned in our previous pilot study delivering treatment services to males [32, 33] was that potential participants were largely unaware of the purposes and goals of research. In the United States, large segments of the population have either participated in or been exposed to behavioral and/or medical research. In contrast, treatment research in Georgia is largely unknown to the population, so an important initial step in the recruitment of potential participants was to ensure that they fully understood the nature of behavioral research and the goals and purposes of the research in which they would be enrolling. Thus, significant effort was expended in our previous treatment research in explaining the concept of informed consent and assuring potential participants regarding confidentiality. This need for a review of the nature and role of informed consent in behavioral research also proved true with our female participants in our Aim 3 study. Informed consent—and assurance of confidentiality—took a minimum for 40 minutes. Most Aim 3 study participants did not wish to leave with a copy of their signed consent form; however, all took the small part of the consent form wherein they were informed of their rights, and information about the IRB was provided. This issue is vitally important to bear in mind when conducting research in a culture whose population is research-naïve.

A second lesson that this project taught us about treatment adaptation is the vital role that mixed methods research plays. The qualitative research conducted in studies 1 and 2 allowed for a greater understanding of the relationship of substance-using women to their Georgia culture, their substance use and sexual practices that put them at risk, and the lack of fit between their treatment needs and the services that were available to them. Findings from these two studies allowed for an integrative RBT+WC approach to treatment that was much more aligned with the needs of the population. Moreover, data from both the qualitative and quantitative interviews with participants in Aim 3 study allowed for a deeper understanding of what outcomes were important to measure and how best to deliver the treatment so that it would have the promise of meaningfully impacting their lives.

Our Aim 4 study now holds the promise of providing meaningful findings regarding feasibility and initial efficacy of a culturally sensitive, women-centered, and comprehensive treatment approach that is aligned with the needs of the intended population. It will represent only the first step in further developing and refining such an approach so that it will prove impactful for substance-abusing women in Georgia.

## Figures and Tables

**Table 1:** Demographic and background characteristics of the Aim 3 study sample (*N* = 20).

	M (SD)	f (%)
Age	34.6 (10.0)	
Education		
Completed secondary education		3 (15)
Some postsecondary education		5 (25)
Completed vocational education (2 years in vocational or community college) or obtained university degree		11 (55)
Some postgraduate study		1 (5)
Unemployed		13 (65)
Experienced serious depression: lifetime		17 (85)
Experienced serious depression: past 30 days		6 (30)
Prescribed a medication for psychological/emotional issues: lifetime		13 (65)
Prescribed a medication for psychological/emotional issues: past 30 days		2 (10)
Lived with your main sex partner		12 (60)
Number of times engaged in sex in the past 30 days	12 (10.1)	
Percent of times unprotected	72.9 (.4)	
Main sex partner has a problem with drugs		10 (50)
Believe that their main sex partner is having sex with someone else		6 (30)
Experienced physical abuse violence: lifetime		9 (45)
Forced to engage in sexual acts against your will		5 (25)

**Table 2:** Model-estimated means and (standard errors) from the generalized estimated equations analyses of urine screening results and needs assessment (*N* = 20).

	Intervention main effect		Time main effect		Interaction effect			
	RBT	Case management	Baseline	End of treatment	RBT condition		Case management condition	
					Baseline	End of treatment	Baseline	End of treatment
Urine screening	.4 (.1)	.6 (.1)	.7 (.1)	.4 (.1)	.6 (.2)	.3 (.2)	.8 (.1)	.4 (.2)
Needs assessment	4.6 (.7)	3.9 (.6)	6.9 (.5)	2.6 (.5)	6.6 (.8)	3.2 (.7)	7.2 (.5)	2.1 (.6)

## Appendix

*Theoretical Foundations of RBT and WC*

*RBT: Treatment Plan and Goals.* Drug abstinence is the primary treatment plan focus. A Functional Assessment determines problem areas associated with drug use and is the basis for other goals. The individualized treatment plan focuses on goals directly related to decreasing/eliminating drug use. The priority of goals is dynamic, based on most pressing issues for drug abstinence initiation and continuation.

*RBT: Reinforcing Small Goals to Reach the Large Goal.* RBT is an active therapeutic approach. Each large treatment plan goal is broken into small steps. Progress of smaller and then larger goal behaviors are graphed at each visit (see below). Active counselor support overcomes “resistance” due to past failures.

*RBT: Density of Alternative Reinforcers.* RBT increases the density of alternative (nondrug) reinforcers in the person's naturalistic environment. Thus, participants complete interest inventories and Functional Assessments with their counselor to determine what activities might serve as positive reinforcers and during periods of previous abstinence what activities or events were functioning as competing alternative reinforcers. Based upon urine tests negative for monitored substances (opioids, amphetamines, benzodiazepines, THC, buprenorphine, and methadone), participants receive reward cards that have monetary value and exchangeable for goods and services.

*RBT: Response to Drug Use and Proactive Outreach.* Having participants provide urine samples twice weekly during treatment maximizes the ability to detect noncompliance when/if it occurs and can prevent lapses from escalating to relapses. A stimulant-positive urine test results in an individual lapse-focused counseling session (e.g., Functional Assessment; FA) and a “time out” from other RBT aspects. A missed RBT session results in proactive counseling outreach procedures that same day.

*RBT: Graphing of Progress.* Behaviors emitted that are congruent or incongruent with goals are graphed by the counselor with the participant. Frequent and consistent graphing of target behaviors helps to focus both counselor and participant on the tasks of treatment and also serves to provide “early warning signs” that precede a lapse or relapse. Graphing is a therapy process and does not constitute outcome measurement.

*RBT: Skills Training in Recreation, Life Goals, and Other Life Skills.* RBT delivers skills training in an individual or group format, with similar efficacy. Skills-training takes the form of recreational activity sampling, social club, and 12 educational modules (each topic is repeated three times during the 12 weeks). Each skill element is manualized; an approach previously found to be acceptable to participants.

*WC: Reductions in Sex Risk and Interpersonal Violence.* Four modules from Women's CoOp were incorporated in RBT+WC. Module 1 educates women about the risks involved in alcohol and drug abuse and how certain sex behaviors increase HIV risk. Module 2 was adapted to focus on the context of sexual risk for women in Georgia and was revised to include information gained in studies 1 and 2 (e.g., stigma, double-standards for men and women in number of sexual partners). During this time, participants are asked to practice the mechanics of the correct use of male and female condoms using penile and vaginal models. Each woman has her own model to work with and has an opportunity to take home male and female condoms and experience them and return the next session to discuss how it felt to insert a female condom if they had never seen or used one. Module 3 teaches participants negotiation skills to be used with male partners and role-playing and rehearsal for practice. It directly addresses fears about intimate partner violence related to forced and unsafe sex practices and sexual negotiation. Module 4 focuses on interpersonal violence prevention, including domestic violence and rape, and strategies for violence prevention. Nonviolent resolutions are presented including a process with steps for “fair fighting” to address conflict resolution.

Because the Women's CoOp modules were a key new component of RBT+WC, it was imperative that their messages were interwoven into RBT rather than having the modules seen as independent, parallel, or add-on. Thus, to integrate this effective HIV prevention into RBT, we reinforced the Women's CoOp messages in the individual counseling sessions by graphing the frequency of safe and unsafe sex acts, discussing condom use and condom protection negotiations, and employing a functional analysis when unprotected sexual acts were reported.
